# Traction‐assisted Endoscopic Retrograde Cholangiopancreatography Using an S‐O Clip for Difficult Biliary Cannulation in Billroth II Reconstruction With Tumor‐associated Distortion

**DOI:** 10.1002/deo2.70386

**Published:** 2026-07-28

**Authors:** Katsuyuki Dainaka, Ryota Ujihara, Tsuyoshi Sawai, Takashi Otsuka, Juichiro Yoshida, Hideki Fujii, Naoya Tomatsuri, Hideki Sato

**Affiliations:** ^1^ Department of Gastroenterology Japanese Red Cross Kyoto Daiichi Hospital Kyoto Japan

**Keywords:** balloon‐assisted ERCP, biliary cannulation, Billroth II reconstruction, malignant anatomical distortion, S‐O clip

## Abstract

Endoscopic retrograde cholangiopancreatography (ERCP) in patients with surgically altered anatomy is technically challenging, especially when the papilla is poorly oriented relative to the endoscope. We report a case of traction‐assisted ERCP using an S‐O clip for difficult biliary cannulation in a patient with Billroth II reconstruction and tumor‐associated distortion. A patient with a history of distal gastrectomy with Billroth II reconstruction for gastric cancer presented with obstructive jaundice caused by recurrent gastric cancer with biliary stricture. During double‐balloon enteroscopy‐assisted ERCP, marked papillary malorientation due to distortion of the afferent limb made standard biliary cannulation difficult. To improve papillary visualization, an S‐O clip was placed on a mucosal fold on the oral side of the papilla, and the loop was anchored to the anal‐side intestinal wall to create clip‐to‐wall traction. This maneuver improved frontal visualization of the papillary orifice and stabilized the biliary axis, allowing successful biliary cannulation with an MTW ERCP catheter and guidewire. Endoscopic sphincterotomy and plastic stent placement were then completed without adverse events. Although similar traction‐assisted ERCP techniques have been reported, this case highlights the usefulness of the method in tumor‐associated distortion, where traction did not dramatically reposition the papilla but provided sufficient frontal visualization for biliary cannulation. Traction‐assisted ERCP may be a useful adjunctive technique in selected cases with altered anatomy and compromised papillary orientation.

## 1 Introduction

Selective biliary cannulation during endoscopic retrograde cholangiopancreatography (ERCP) in patients with surgically altered anatomy remains technically demanding [1]. In particular, biliary cannulation becomes difficult when the papilla does not face the endoscope because of postoperative adhesion, diverticular deformity, or tumor‐associated distortion. Traction‐assisted techniques using clip devices have been reported as rescue methods for difficult biliary cannulation. However, reports describing their use in Billroth II reconstruction complicated by tumor‐associated distortion remain limited. We report a case of successful traction‐assisted ERCP using an S‐O clip in a patient with Billroth II reconstruction and recurrent gastric cancer causing afferent limb distortion.

## 2 Case Report

A patient with a history of distal gastrectomy with Billroth II reconstruction for gastric cancer was admitted with obstructive jaundice. Imaging studies suggested recurrent gastric cancer with distal biliary stricture. Double‐balloon enteroscopy‐assisted ERCP was performed using a double‐balloon endoscope (EN‐840T; Fujifilm, Tokyo, Japan) fitted with a transparent distal cap.

**FIGURE 1:**
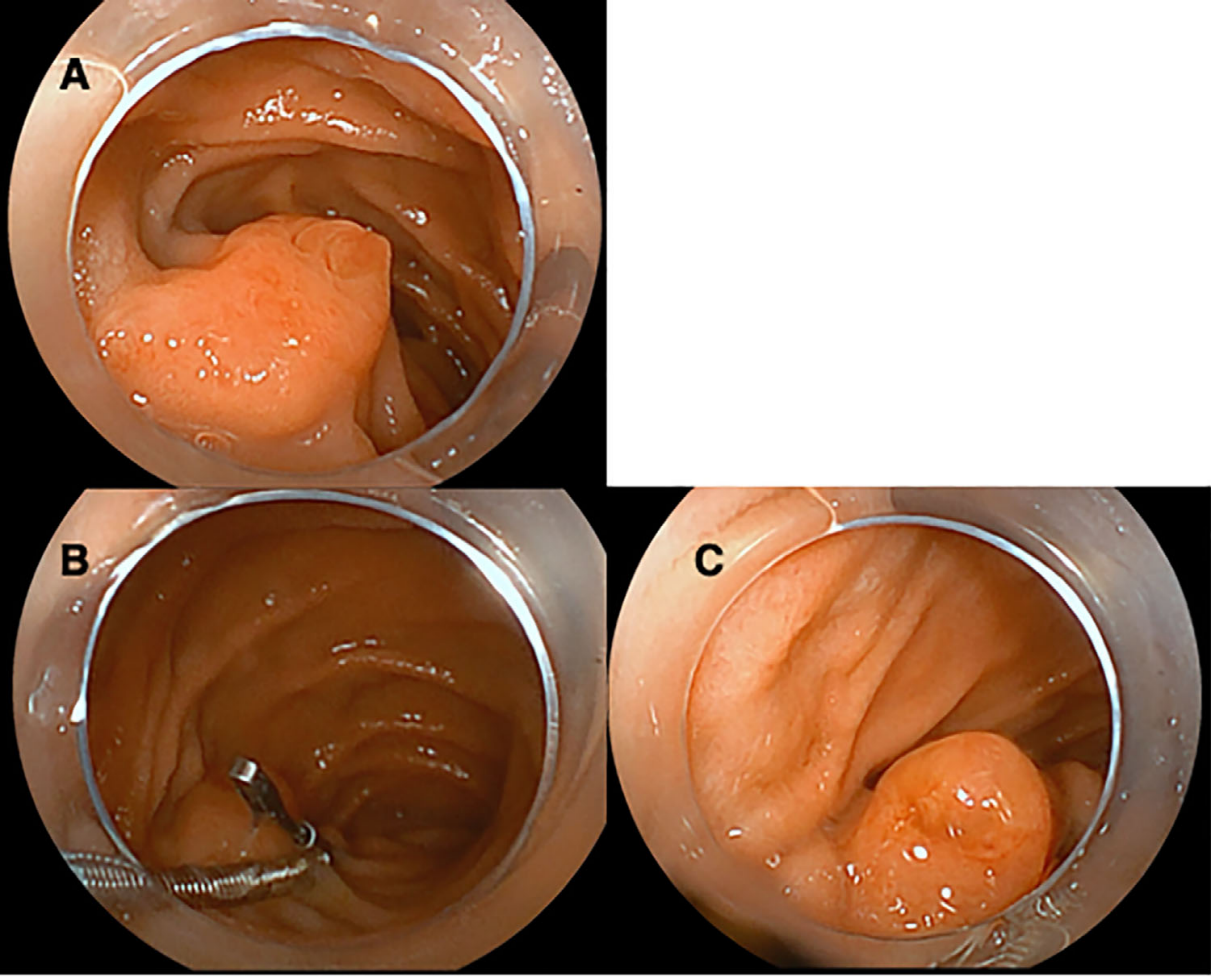
Effect of traction on papillary orientation during endoscopic retrograde cholangiopancreatography (ERCP). (A) The papilla was slightly deviated away from the endoscope and showed limited mobility with suction, making selective biliary cannulation difficult. (B) The loop of the S‐O clip placed on the oral side of the papilla was anchored to the anal‐side intestinal wall, creating clip‐to‐wall traction over an approximate distance of 5 cm. (C) After traction, a stable frontal view of the papilla was obtained, allowing successful biliary cannulation.

Endoscopic examination revealed marked distortion of the afferent limb. The papilla was maloriented and deviated away from the endoscope, with limited mobility on suction, making selective biliary cannulation difficult. To improve papillary visualization, traction was applied using an S‐O clip (Zeon Medical, Tokyo, Japan). The clip was placed on a mucosal fold on the oral side of the papilla, and its loop was anchored to the anal‐side intestinal wall, creating clip‐to‐wall traction over an estimated distance of approximately 5 cm under fluoroscopic guidance. This configuration reoriented the papilla toward the endoscope while preserving the working channel and maintaining a stable frontal view for cannulation. This maneuver improved frontal visualization of the papillary orifice and partially corrected the biliary axis (Figure 1).

Selective biliary cannulation was subsequently achieved using an MTW ERCP catheter (ABIS, Hyogo, Japan) and a 0.025‐inch guidewire (Endoselector; Boston Scientific, Marlborough, MA, USA). Endoscopic sphincterotomy was then performed, followed by successful placement of a plastic biliary stent (REGULS; Japan Lifeline, Tokyo, Japan). No adverse events occurred during or after the procedure. In addition, no obvious deterioration in gastrointestinal peristalsis or endoscope stability was observed during traction. The video of the procedure is shown in Video S1 Supporting Information.

## 3 Discussion

Traction‐assisted ERCP using clip devices is a clinically relevant rescue technique for difficult biliary cannulation, particularly in patients with surgically altered anatomy [2, 3, 4, 5, 6, 7]. Previous reports have demonstrated the usefulness of the S‐O clip in periampullary diverticulum and balloon enteroscopy‐assisted ERCP [2, 3]. Suda et al. also reported an S‐O clip‐assisted technique for biliary cannulation in Billroth II reconstruction. In that case, traction resolved postoperative bile duct kinking and led to marked repositioning of the papilla [4].

The present case differs in that the difficulty in biliary cannulation was primarily caused by tumor‐associated distortion of the afferent limb rather than postoperative bile duct kinking. In our case, traction did not result in a dramatic positional change of the papilla. Instead, its main benefit was stabilization of the papillary axis and improvement of frontal visualization, which were sufficient to achieve successful biliary cannulation. Thus, the clinical value of traction in this setting may lie not only in repositioning the papilla itself but also in providing stable axis correction for cannulation.

**FIGURE 2:**
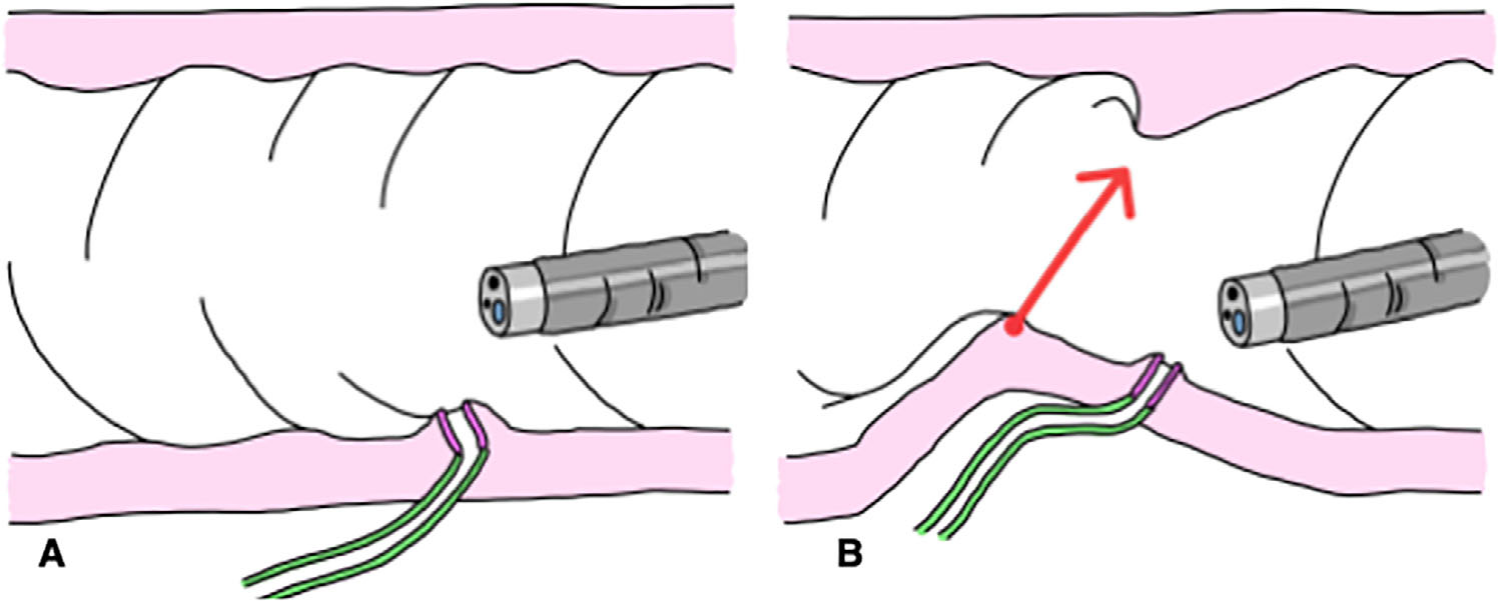
Mechanism of traction‐assisted endoscopic retrograde cholangiopancreatography (ERCP) using an S‐O clip. (A) Papillary malorientation before traction makes selective biliary cannulation difficult. (B) Placement of an S‐O clip on the oral side of the papilla, followed by clip‐to‐wall traction toward the anal side, reorients and stabilizes the papillary axis toward the endoscope, allowing a stable frontal view for biliary cannulation.

In the present method, the clip was placed on the oral side of the papilla and anchored to the anal‐side intestinal wall. This clip‐to‐wall traction allowed reorientation of the papilla toward the endoscope while preserving the working channel and without obvious interference with endoscopic maneuvers. No obvious deterioration in gastrointestinal peristalsis or endoscope stability was observed during traction(Figure 2).

This technique is not required in all patients with Billroth II reconstruction. Rather, it appears most useful when papillary orientation is compromised, and standard endoscopic maneuvers fail to obtain a stable frontal view [1, 4]. Although this technique does not eliminate the need for operator experience, stabilization of the papillary axis may help reduce the technical difficulty of biliary cannulation in selected difficult cases, including those encountered by less experienced endoscopists.

## Author Contributions

**Katsuyuki Dainaka**: conceptualization, procedure, data curation, video editing, and writing – original draft. **Ryota Ujihara**: figure preparation, procedure support, and review and editing. **Tsuyoshi Sawai**: procedure support and review and editing. **Takashi Otsuka**: procedure support and review and editing. **Juichiro Yoshida**: supervision and review and editing. **Hideki Fujii**: clinical management and review and editing. **Naoya Tomatsuri**: review and editing. **Hideki Sato**: supervision and review and editing.

## Funding

The authors have nothing to report.

## Conflicts of Interest

The authors declare no conflicts of interest.

4

## Supporting information



Traction‐assisted endoscopic retrograde cholangiopancreatography (ERCP) using an S‐O clip in a patient with Billroth II reconstruction. Tumor‐associated distortion of the afferent limb caused papillary malorientation, making biliary cannulation difficult. Placement of an S‐O clip on the oral side of the papilla, followed by clip‐to‐wall traction toward the anal side, improved frontal visualization of the papilla and stabilized the biliary axis, allowing successful biliary cannulation and plastic stent placement.
